# Transcriptional control of intestinal cholesterol absorption, adipose energy expenditure and lipid handling by Sortilin

**DOI:** 10.1038/s41598-018-27416-y

**Published:** 2018-06-13

**Authors:** Sumihiko Hagita, Maximillian A. Rogers, Tan Pham, Jennifer R. Wen, Andrew K. Mlynarchik, Masanori Aikawa, Elena Aikawa

**Affiliations:** 1 Center for Interdisciplinary Cardiovascular Sciences, Cardiovascular Division, Brigham and Women’s Hospital, Harvard Medical School, Boston, MA 02115 USA; 2 Center for Excellence in Vascular Biology, Cardiovascular Division, Brigham and Women’s Hospital, Harvard Medical School, Boston, MA 02115 USA

## Abstract

The sorting receptor Sortilin functions in the regulation of glucose and lipid metabolism. Dysfunctional lipid uptake, storage, and metabolism contribute to several major human diseases including atherosclerosis and obesity. Sortilin associates with cardiovascular disease; however, the role of Sortilin in adipose tissue and lipid metabolism remains unclear. Here we show that in the low-density lipoprotein receptor-deficient (Ldlr^−/−^) atherosclerosis model, Sortilin deficiency (Sort1^−/−^) in female mice suppresses Niemann-Pick type C1-Like 1 (Npc1l1) mRNA levels, reduces body and white adipose tissue weight, and improves brown adipose tissue function partially via transcriptional downregulation of Krüppel-like factor 4 and Liver X receptor. Female Ldlr^−/−^Sort1^−/−^ mice on a high-fat/cholesterol diet had elevated plasma Fibroblast growth factor 21 and Adiponectin, an adipokine that when reduced is associated with obesity and cardiovascular disease-related factors. Additionally, Sort1 deficiency suppressed cholesterol absorption in both female mice *ex vivo* intestinal tissue and human colon Caco-2 cells in a similar manner to treatment with the NPC1L1 inhibitor ezetimibe. Together our findings support a novel role of Sortilin in energy regulation and lipid homeostasis in female mice, which may be a potential therapeutic target for obesity and cardiovascular disease.

## Introduction

Dysfunctional lipid handling and metabolism are associated with obesity and metabolic disease^1,2^, which in turn are major risk factors for cardiovascular disease^3^. Suppression of factors associated with obesity and metabolic disease may therefore reduce the development of cardiovascular disease, which has high unmet medical needs. Excess adipose tissue results in obesity. Adipose tissue is characterized as white adipose tissue (WAT), which functions as lipid storage, and brown adipose tissue (BAT) that participates in lipid metabolism and energy expenditure^4–8^. Further connecting cardiovascular disease to dysfunctional lipid metabolism is the intestinal cholesterol absorption-related protein, Niemann-Pick type C1-Like 1 (NPC1L1). NPC1L1 is the target of the hypercholesterolemia drug ezetimibe^9^. Supporting a role of intestinal cholesterol absorption in adipose tissue and cardiovascular disease, ezetimibe treatment or Npc1l1 deficiency in mice on a high-fat diet, reduces body weight and adipogenesis^9,10^.

The sorting protein Sortilin functions as both a receptor and trafficking molecule with both intracellular and extracellular regulatory functions^11,12^. Genome wide association studies have connected the 1p13 locus harboring the Sortilin encoding gene, SORT1 to plasma low-density lipoprotein (LDL) cholesterol, myocardial infarction, aortic aneurysm, and coronary artery calcification^13–16^. Sortilin has been associated with atherosclerosis, very-low density lipoprotein secretion, macrophage LDL uptake and foam cell formation, inflammatory cytokine secretion, and hepatic steatosis^17–21^. Additionally, we recently established a mechanistic role of Sortilin in the loading of extracellular vesicles contributing to cardiovascular calcification^22^, along with associating elevated serum Sortilin to aortic calcification and cardiovascular risk^23^.

While the association of Sortilin to cardiovascular disease is established, whether Sortilin plays a role in adipose tissue function, body weight gain, and factors shared by both cardiovascular disease and obesity, like NPC1L1, is unclear. Conflicting results have been reported for Sortilin in diet-induced obesity models generated by different methods. Sort1 deficiency generated by deleting 41 codons in exon 14, resulting in a reading frame disruption, reduced body weight gain and visceral fat in diet-induced obesity male C57BL/6 mice; without altering viability, fertility, or showing any gross abnormalities^21^. In contrast, Sort1 deficiency generated by introducing a stop codon into the second intron of the Sort1 gene altered adipose glucose metabolism but had no effect on diet-induced obesity in male C57BL/6 mice^24^. Given the strong connection of Sortilin to cardiovascular disease, we sought to assess if under atherosclerosis-related conditions, Sort1 deficiency alters adipose tissue function, weight gain, and NPC1L1-mediated cholesterol absorption in male and female mice.

## Results

### Sort1 deficiency reduced female Ldlr^−/−^ mouse body and WAT weight

**Figure 1:**
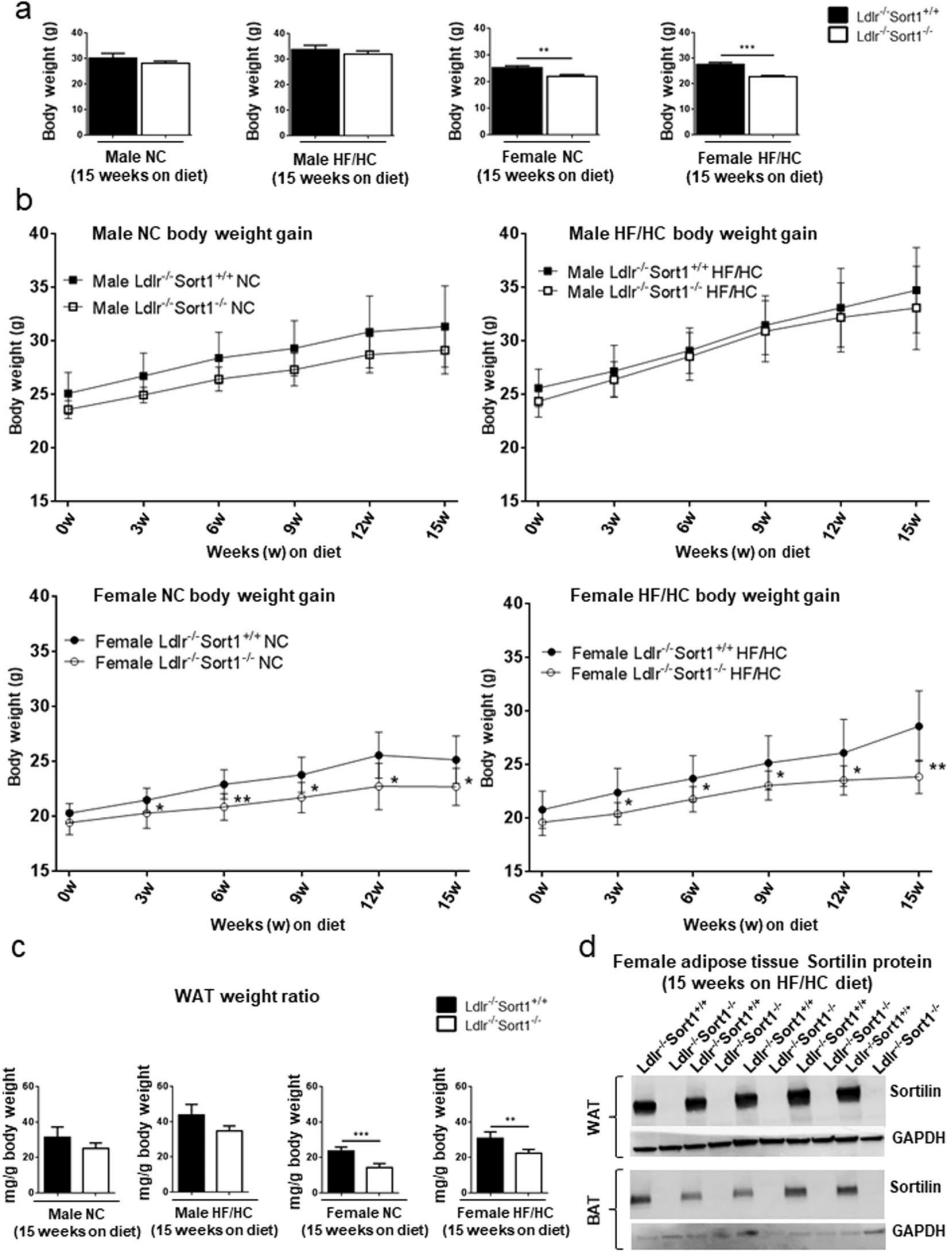
Sort1 deficiency reduced body and WAT weight in female Ldlr^−/−^ mice. (**a**) Body weight in grams (g), (**b**) body weight gain, and (**c**) WAT weight ratio for Ldlr^−/−^Sort1^+/+^ and Ldlr^−/−^Sort1^−/−^ mice after 15 weeks (15w) on normal chow (NC) or high-fat/cholesterol diet (HF/HC); (n = 5–10 mice/group). (**d**) Confirmation of Sortilin protein deficiency in 15-week HF/HC-diet fed female Sort1^−/−^ mice WAT and brown adipose tissue (BAT); (n = 5 mice/group). GAPDH loading controls were run on the same blot, and the membrane was cut prior to incubating with primary antibody. Full-length blots are presented in Supplemental Fig. S4a and b. *P < 0.05, **P < 0.01, ***P < 0.001 versus sex and diet matched Ldlr^−/−^Sort1^+/+^ mice, analyzed by t-test; values are presented as mean ± SEM.

As obesity and metabolic dysfunction are cardiovascular risk factors, we assessed whether Sort1 deficiency altered adipose tissue in atherosclerotic mice. Sort1-deficient mice (Sort1^−/−^) were generated by targeted deletion of 191 base pairs of exon 14 and crossed to C57BL/6 Ldlr-deficient mice^22^. To examine the effects of Sort1 deficiency on body weight, 10-week-old male and female Ldlr^−/−^Sort1^+/+^ and Ldlr^−/−^Sort1^−/−^ mice were fed either normal chow (NC) or high-fat/cholesterol (HF/HC) diet for 15 weeks. Female Ldlr^−/−^Sort1^−/−^ mice on both NC and HF/HC diet had significantly lower body weight gain starting at three-weeks-in that was maintained through the remainder of the 15-week feeding (Fig. 1a,b). Male Ldlr^−/−^Sort1^−/−^ mice body weight was not different from controls (Fig. 1a,b). Explaining the reduced weight gain, female Ldlr^−/−^Sort1^−/−^ mice had lower WAT weight on both NC and HF/HC diets, compared to Ldlr^−/−^Sort1^+/+^ mice (Fig. 1c). Sort1 deficiency in adipose tissue was confirmed by mRNA levels (Supplementary Fig. S1a,b) and by western blot protein abundance measurements (Fig. 1d). Food consumption, liver weight, plasma glucose, and hepatic lipid contents were not altered between Ldlr^−/−^Sort1^+/+^ and Ldlr^−/−^Sort1^−/−^ female mice on a 15-week HF/HC diet (Supplementary Table S1).

### Sort1 deficiency reduced WAT adipocyte size, and increased Adiponectin in HF/HC fed female Ldlr^−/−^ mice

**Figure 2:**
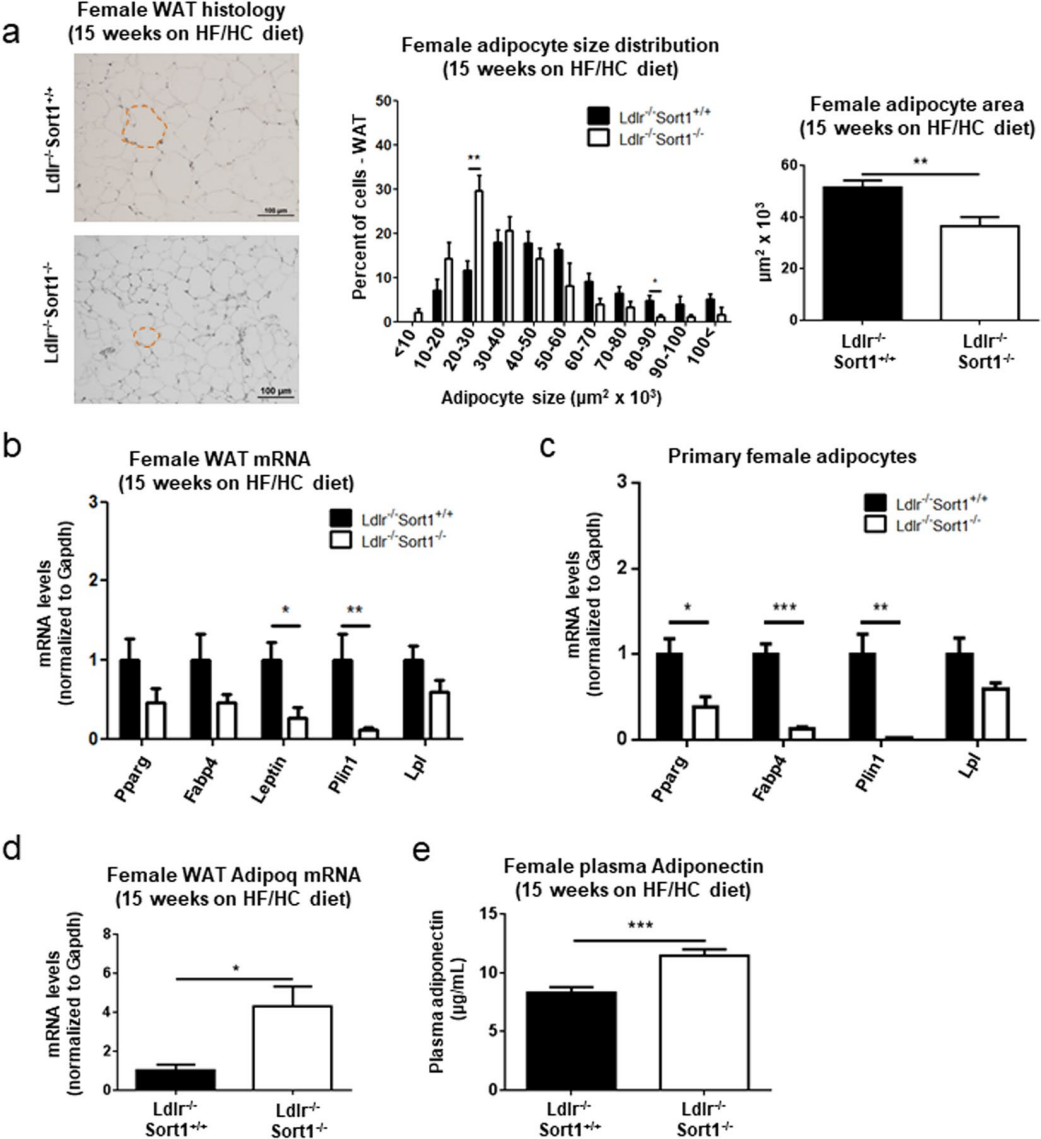
Sort1 deficiency impaired WAT formation in female Ldlr^−/−^ mice fed a HF/HC diet. (**a**) Representative hematoxylin and eosin stained sections (example of a white adipocyte outlined by dashed red line; scale bars indicate 100 μm), quantified adipocyte size distribution, and average size of white adipose tissue (WAT) adipocytes in female Ldlr^−/−^Sort1^+/+^ and Ldlr^−/−^Sort1^−/−^ mice fed a high-fat/cholesterol (HF/HC) diet for 15 weeks; (n = 5–6 mice/group). (**b**) Female mice on a HF/HC diet for 15 weeks mRNA levels of genes related to white adipocyte formation in WAT, and in (**c**) primary white adipocytes differentiated from female mouse WAT; (n = 3–6 mice/group). (**d**) WAT Adipoq mRNA levels (n = 5–6 mice/group), and (**e**) plasma Adiponectin protein concentration in female Ldlr^−/−^Sort1^+/+^ and Ldlr^−/−^Sort1^−/−^ mice fed a HF/HC diet for 15 weeks; (n = 10 mice/group). *P < 0.05, **P < 0.01, ***P < 0.001 versus Ldlr^−/−^Sort1^+/+^, analyzed by t-test; values are presented as mean ± SEM.

Hematoxylin and eosin staining of WAT from 15-week HF/HC-fed female mice revealed smaller adipocytes in Sort1-deficient mice compared to Ldlr^−/−^Sort1^+/+^ mice (Fig. 2a). Real-time PCR analysis showed white adipocyte-related genes including, Leptin and Plin1, had significantly decreased mRNA levels in female Ldlr^−/−^Sort1^−/−^ mouse WAT, compared to Ldlr^−/−^Sort1^+/+^ mice on a 15-week HF/HC diet (Fig. 2b). Primary adipocytes differentiated from female Ldlr^−/−^Sort1^−/−^ mouse WAT had reduced mRNA levels of the adipose tissue-related genes Pparg, Fabp4, and Plin1 (Fig. 2c). Similar mRNA level reductions were observed in WAT from NC-fed female Ldlr^−/−^Sort1^−/−^ mice, but not in 15-week HF/HC-diet fed male mice (Supplementary Fig. S1c,f). As adipocytes secret Adiponectin, an adipokine that when reduced is associated with obesity and cardiovascular risk factors in animal models and humans^25^, we assessed Adiponectin levels in female Ldlr^−/−^Sort1^−/−^ mice. Increased mRNA levels of the Adiponectin encoding gene, Adipoq were observed in 15-week HF/HC-fed Ldlr^−/−^Sort1^−/−^ mouse WAT (Fig. 2d). Plasma Adiponectin protein abundance was similarly increased in 15-week HF/HC-fed female Ldlr^−/−^Sort1^−/−^ mice (Fig. 2e). Adipoq mRNA levels and plasma Adiponectin protein abundance was not significantly changed in NC-fed female mice, or male Ldlr^−/−^Sort1^−/−^ mice on a 15-week HF/HC diet (Supplementary Fig. S1d,e,g,h).

### Sort1 deficiency increased BAT function in HF/HC-fed female Ldlr^−/−^ mice

**Figure 3:**
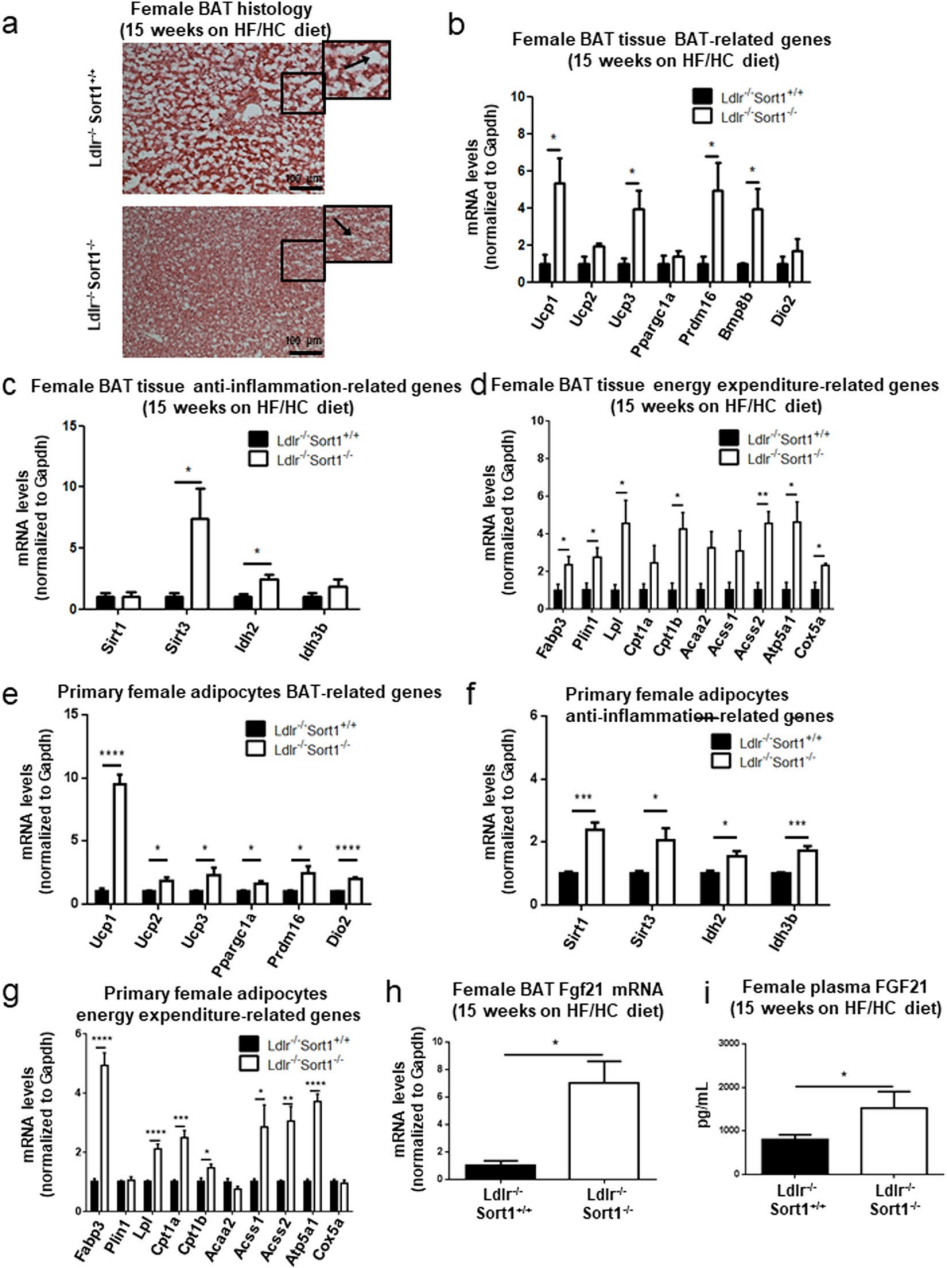
Sort1 deficiency improved BAT function in female Ldlr^−/−^ mice on a HF/HC diet. (**a**) Representative hematoxylin and eosin stained sections of brown adipose tissue (BAT; magnified portions in black boxes show lipid droplets, white areas, indicated by arrows; scale bars indicate 100 μm), and mRNA levels of (**b**) BAT-related, (**c**) anti-inflammation-related, and (**d**) energy expenditure-related genes, including fatty acid utilization, β-oxidation, tricarboxylic acid (TCA) cycle and electron transport chain in BAT of female Ldlr^−/−^Sort1^+/+^ and Ldlr^−/−^Sort1^−/−^ mice fed a high fat/high cholesterol (HF/HC) diet for 15 weeks; (n = 3–4 mice/group). mRNA levels of (**e**) BAT-related, (**f**) anti-inflammation-related, and (**g**) energy expenditure-related genes in primary brown adipocytes differentiated from female mouse BAT; (n = 6). (**h**) Fgf21 expression in BAT, and (**i**) plasma FGF21 concentration in female mice fed a HF/HC diet for 15 weeks; (n = 3–4 mice/group). *P < 0.05, **P < 0.01, ***P < 0.001, ****P < 0.0001 vs. Ldlr^−/−^Sort1^+/+^, analyzed by t-test; values are presented as mean ± SEM.

Sort1 deficiency reduced lipid droplet size, and increased mRNA levels of Ucp1 and other brown adipose-related genes in 15-week HF/HC-diet-fed female Ldlr^−/−^ mice BAT (Fig. 3a,b). To investigate the effects of Sort1 deficiency on BAT function, we measured the mRNA levels of genes related to inflammation and fatty acid utilization in BAT thermogenesis^26–30^. Female Ldlr^−/−^Sort1^−/−^ mice on a 15-week HF/HC diet had elevated mRNA levels of Sirt3 and its regulating oxidoreductase encoding gene, Idh2 (Fig. 3c). Sort1 deficiency additionally increased Fabp3, Plin1, and Lpl mRNA levels in 15-week HF/HC-diet fed female mice; suggesting higher fatty acid utilization and lipolysis in female Ldlr^−/−^ BAT (Fig. 3d). The β-oxidation gene, Cpt1b, the TCA cycle gene Acss2, and electron transport chain genes, Atp5a1 and Cox5a, were increased by Sort1 deficiency in female Ldlr^−/−^ BAT following a 15-week HF/HC diet (Fig. 3d); suggesting increased utilization of fatty acid for energy production in mitochondria. Similar transcriptional effects mediated by Sort1 deficiency were observed in primary adipocytes differentiated from pre-adipocytes isolated from 15-week HF/HC (Fig. 3e–g) and NC-fed female mouse BAT (Supplementary Fig. S2f–h). 15-week HF/HC fed male Ldlr^−/−^Sort1^−/−^ mice did not exhibit improved BAT function (Supplementary Fig. S2a–c). In female Ldlr^−/−^ mice on a 15-week HF/HC diet, Sort1 deficiency increased the mRNA levels (Fig. 3h) and plasma protein abundance (Fig. 3i) of FGF21, a growth factor involved in Adiponectin secretion and BAT function^31^. In 15-week HF/HC-fed male Ldlr^−/−^Sort1^−/−^ mice, no changes were observed in Fgf21 mRNA levels or plasma protein abundance (Supplementary Fig. S2d,e). NC-fed female Ldlr^−/−^Sort1^−/−^ mice did not have significantly increased Fgf21 levels (Supplementary Fig. S2i,j).

### Sort1 deficiency reduced intestinal Npc1l1 mRNA and cholesterol absorption

**Figure 4:**
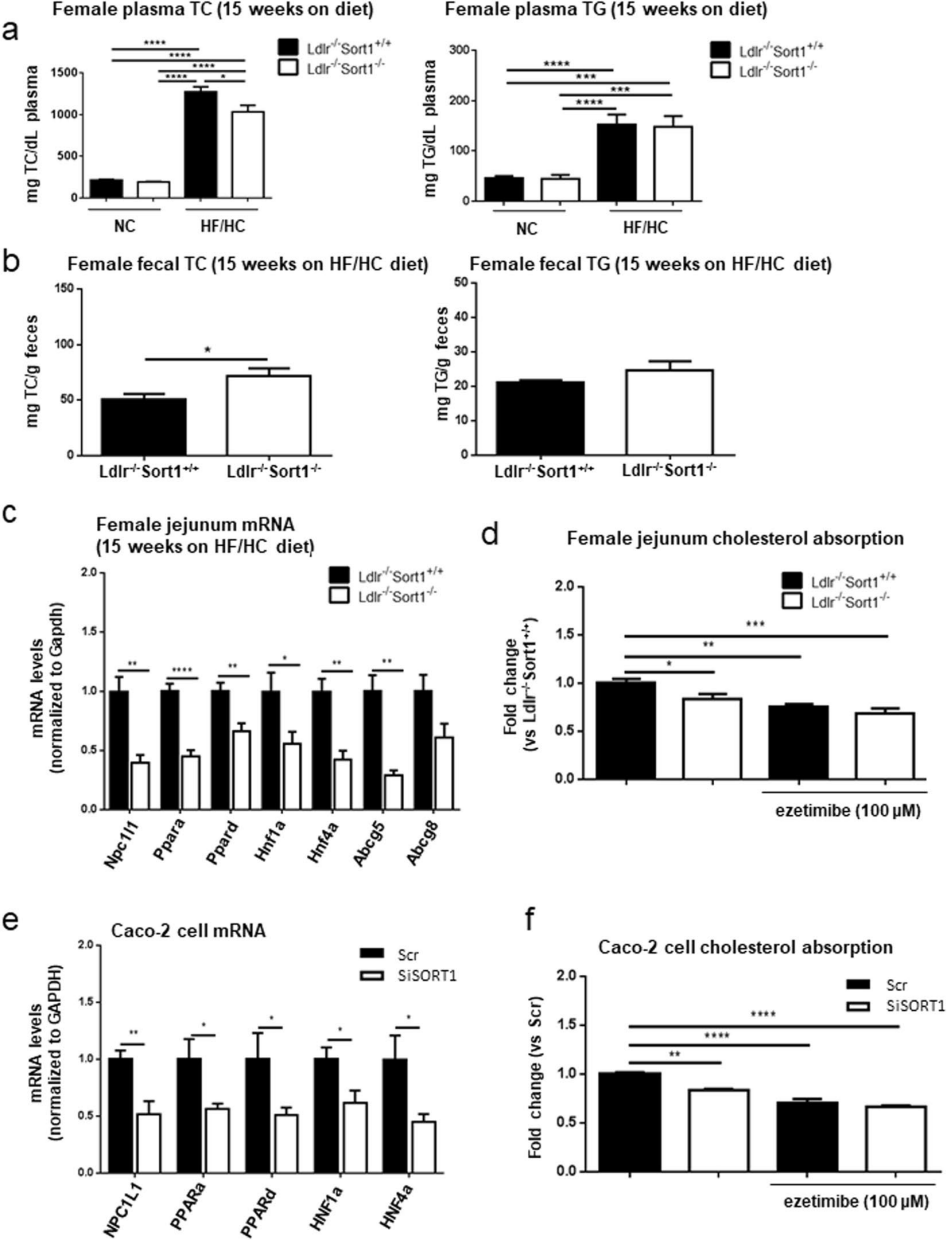
Sort1 deficiency reduced Npc1l1 and plasma cholesterol absorption in murine jejunum and human Caco-2 cells. (**a**) 15-week NC or HF/HC-fed female mice plasma total cholesterol (TC) and triglyceride (TG) levels (n = 8–10 mice/group), and (**b**) fecal TC and TG levels in female Ldlr^−/−^Sort1^+/+^ and Ldlr^−/−^Sort1^−/−^ mice fed a HF/HC diet for 15 weeks (n = 5–6 mice/group). (**c**) Npc1l1, Npc1l1 regulators, and cholesterol efflux genes mRNA levels in female mouse jejunum (n = 6 mice/group). (**d**) *ex vivo* fluorescent cholesterol absorption analysis in mouse jejunum treated with or without ezetimibe; (n = 6–7 mice/group). (**e**) mRNA levels of NPC1L1 and NPC1L1 regulators (n = 6), and (**f**) cholesterol absorption in Caco-2 cells with SORT1 RNA interference (siSORT1) or scrambled control (Scr), with or without ezetimibe treatment; (n = 3). *P < 0.05, **P < 0.01, ***P < 0.001, ****P < 0.0001 versus Ldlr^−/−^Sort1^+/+^ or Scr; analyzed by t-test, except the TC and TG data in panel 4a that were analyzed by ANOVA with Tukey’s multiple comparisons test, and cholesterol absorption data in panels 4d and 4 f that were analyzed by ANOVA with Dunnett’s multiple comparisons test; values are presented as mean ± SEM.

As cholesterol absorption is associated with adipose tissue and weight gain, we assessed whether Sort1 deficiency altered Npc1l1 mRNA levels and intestinal cholesterol absorption in female Ldlr^−/−^ mice. 15-week HF/HC diet significantly increased plasma total cholesterol (TC) and triglycerides (TG) compared to NC diet in female mice (Fig. 4a). Female Ldlr^−/−^Sort1^−/−^ mice had reduced plasma TC on a 15-week HF/HC diet (Fig. 4a). Suggesting impaired cholesterol absorption, fecal lipid contents from individually housed female Ldlr^−/−^Sort1^−/−^ mice had elevated TC compared to Ldlr^−/−^Sort1^+/+^ mice, while fecal TG was unchanged in Sort1-deficient females on a 15-week HF/HC diet (Fig. 4b). To determine the effect of Sort1 deficiency on intestinal cholesterol absorption, we examined the intestinal cholesterol transporter gene, Npc1l1 and its regulators including Ppara, Ppard, Hnf1a, and Hnf4a^32–35^. We also performed a fluorescent cholesterol absorption assay in jejunum of female Ldlr^−/−^Sort1^+/+^ and Ldlr^−/−^Sort1^−/−^ mice. Sort1 deficiency significantly decreased Npc1l1, Ppara, Ppard, Hnf1a, and Hnf4a mRNA levels in female Ldlr^−/−^ mice jejunum (Fig. 4c). Jejunum Abcg5 mRNA levels were reduced, and Abcg8 mRNA levels were lower but did not reach significance in 15-week HF/HC-fed Sort1-deficient female mice (Fig. 4c); suggesting reduced intestinal cholesterol uptake and possibly reduced efflux in Sort1-deficient female mice. Sort1 deficiency or ezetimibe treatment reduced *ex vivo* female mice jejunum cholesterol absorption (Fig. 4d). Reduced NPC1L1 mRNA levels and cholesterol absorption were also observed in human colon Caco-2 cells with SORT1 RNA interference (Fig. 4e,f).

### Sort1 deficiency reduced WAT, BAT, and intestinal LXR-mediated transcription

**Figure 5:**
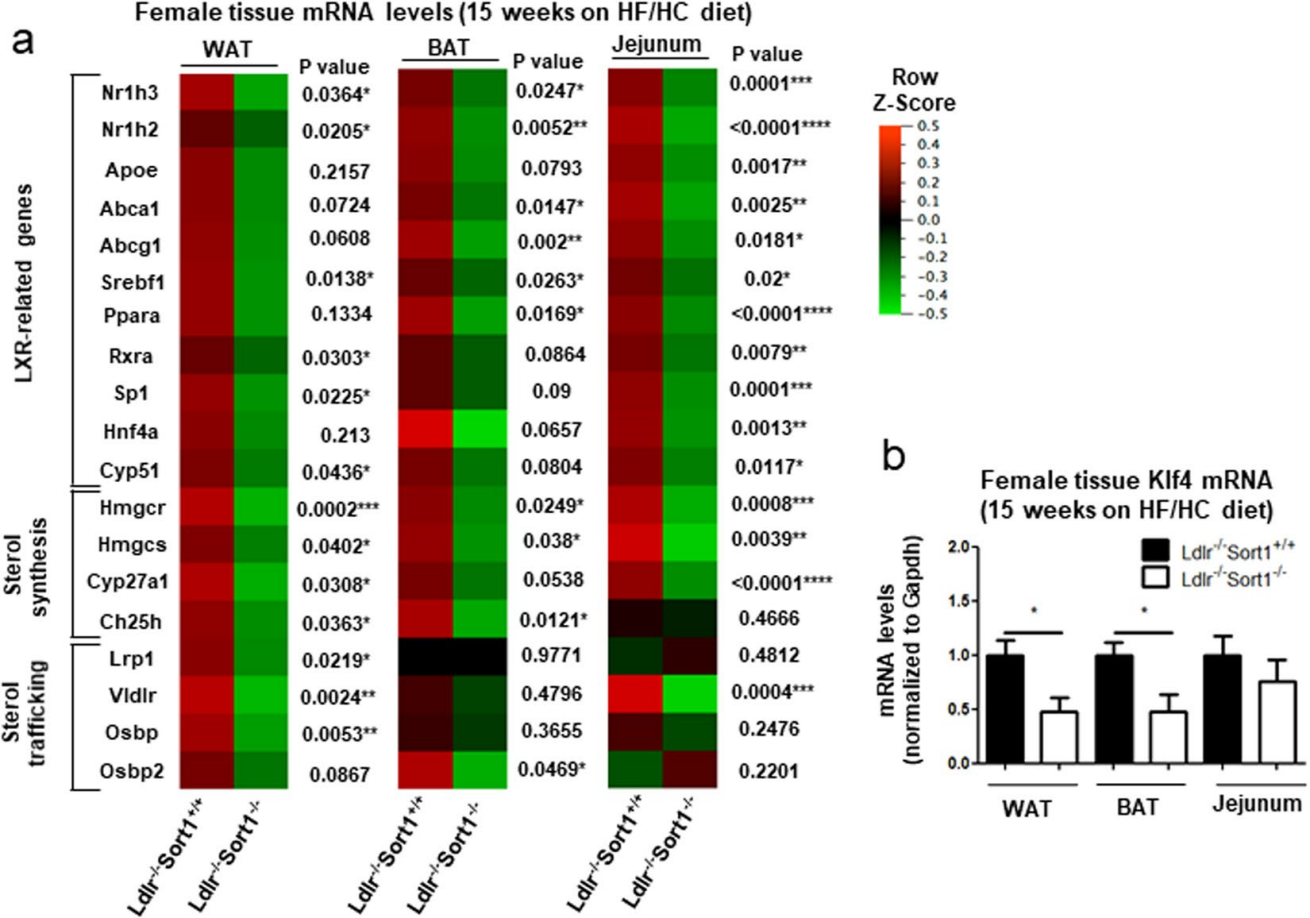
Sort1 deficiency decreased LXR-related transcription in female Ldlr^−/−^ mice on a HF/HC diet. (**a**) Heat maps of LXR-related, sterol synthesis-related, and sterol trafficking-related genes mRNA levels in WAT, BAT, and jejunum tissue of female mice fed a HF/HC diet for 15 weeks (P value shown for each gene; data also presented as bar graphs in Supplemental Fig. S3); (n = 5–6 mice/group). (**b**) Klf4 mRNA levels in WAT, BAT, and jejunum of female mice on a HF/HC diet for 15 weeks; (n = 6 mice/group). *P < 0.05, **P < 0.01, ***P < 0.001, ****P < 0.0001 vs. Ldlr^−/−^Sort1^+/+^, analyzed by t-test; values are presented as mean ± SEM.

**Figure 6:**
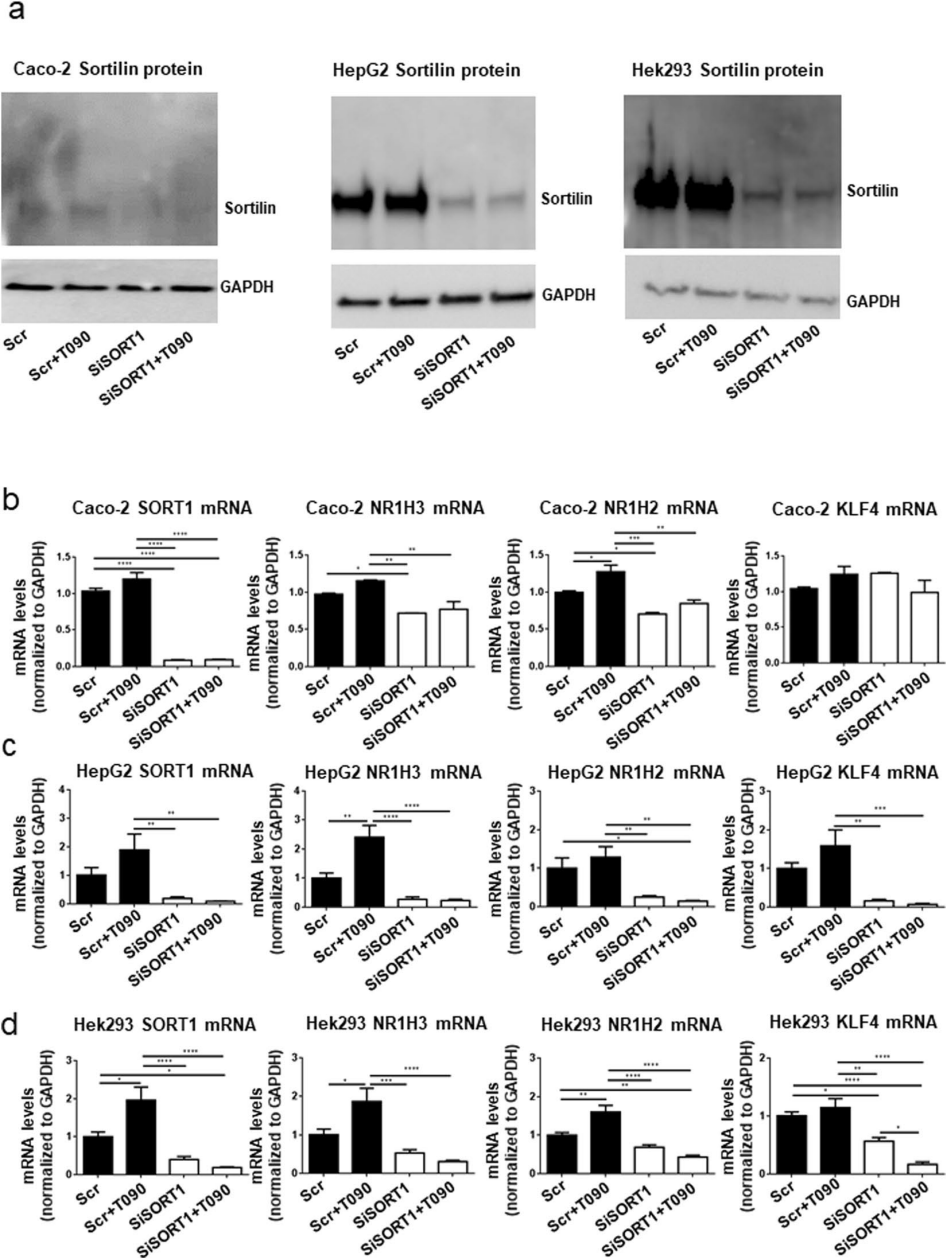
SORT1 deficiency suppressed LXR-mediated transcription in human Caco-2, HepG2, and Hek293 cells. (**a**) Sortilin protein reduction confirmation in Caco-2, HepG2, and Hek293 cells following SORT1 RNA interference (siSort1) or scrambled control (Scr), with or without 24-hour 1 μM T0901317 (T090) LXR agonist treatment; (n = 3; representative western blots shown). GAPDH loading controls were run on the same blot, and the membrane was cut prior to incubating with primary antibody. Full-length blots are presented in Supplemental Fig. S4c. (**b**) Caco-2, (**c**) HepG2, and (**d**) Hek293 cells SORT1, LXRα/β (NR1H3, NR1H2), and KLF4 mRNA levels following SORT1 RNA interference, with or without 24-hour 1 μM T090 treatment; (n = 3–6). *P < 0.05, **P < 0.01, ***P < 0.001, ****P < 0.0001 vs. Scr control, analyzed by ANOVA with Tukey’s multiple comparisons test; values are presented as mean ± SEM.

As Sort1 deficiency transcriptionally regulated WAT and BAT function, along with jejunum intestinal cholesterol absorption, we examined a key transcriptional lipid metabolism modulator, LXRα/β in female mice fed a 15-week HF/HC diet. Sort1 deficiency reduced mRNA levels of LXRα/β (Nr1h3 and Nr1h2), LXR-related genes (Rxra, Cyp51, Srebf1, Sp1), sterol synthesis and metabolism genes (Hmgcr, Hmgcs1, Cyp27a1, Ch25h), and sterol trafficking genes (Lrp1, Vldlr, Osbp) in 15-week HF/HC-fed female mice WAT (Fig. 5a and Supplementary Fig. S3a). In 15-week HF/HC-fed female mice BAT, Sort1 deficiency reduced mRNA levels of LXR-related genes (Nr1h3, Nr1h2, Abca1, Abcg1, Srebf1, Ppara), and sterol metabolism and trafficking genes (Hmgcr, Hmgcs1, Ch25h, Osbp2) (Fig. 5a and Supplementary Fig. S3b). 15-week HF/HC-fed female Ldlr^−/−^Sort1^−/−^ mouse jejunum had reduced LXR-related (Nr1h3, Nr1h2, Rxra, Apoe, Abca1, Abcg1, Srebf1, Ppara, Sp1, Hnf4a) and sterol-related (Hmgcr, Hmgcs1, Cyp27a1, Vldlr) mRNA levels (Fig. 5a and Supplementary Fig. S3c). As a recent study demonstrated Krüppel-like factor 4 (Klf4) is a key cardiovascular LXR regulator^36^, we assessed Klf4 levels in Sortilin-deficient mice. Klf4 mRNA levels were significantly reduced in the WAT and BAT, but not jejunum in 15-week HF/HC-fed Ldlr^−/−^Sort1^−/−^ mice compared to Ldlr^−/−^Sort1^+/+^ female mice (Fig. 5b). Further implicating Sortilin as an upstream regulator of LXR-mediated transcription in some cell types, SORT1 RNA interference inhibited LXR agonist, T0901317-mediated induction of LXR (NR1H3, NR1H2) mRNA levels in human colon Caco-2 cells (Fig. 6a,b), the human hepatocyte-like cell line, HepG2 (Fig. 6a,c), and in the human embryonic kidney cell line, Hek293 (Fig. 6a,d). SORT1 deficiency reduced KLF4 mRNA levels in HepG2 (Fig. 6c) and Hek293 cells (Fig. 6d) like Sort1-deficient female mice WAT and BAT (Fig. 5b); however, like Sort1-deficient female mice jejunum (Fig. 5b), SORT1 deficiency did not reduce KLF4 mRNA levels in Caco-2 cells (Fig. 6b).

## Discussion

**Figure 7:**
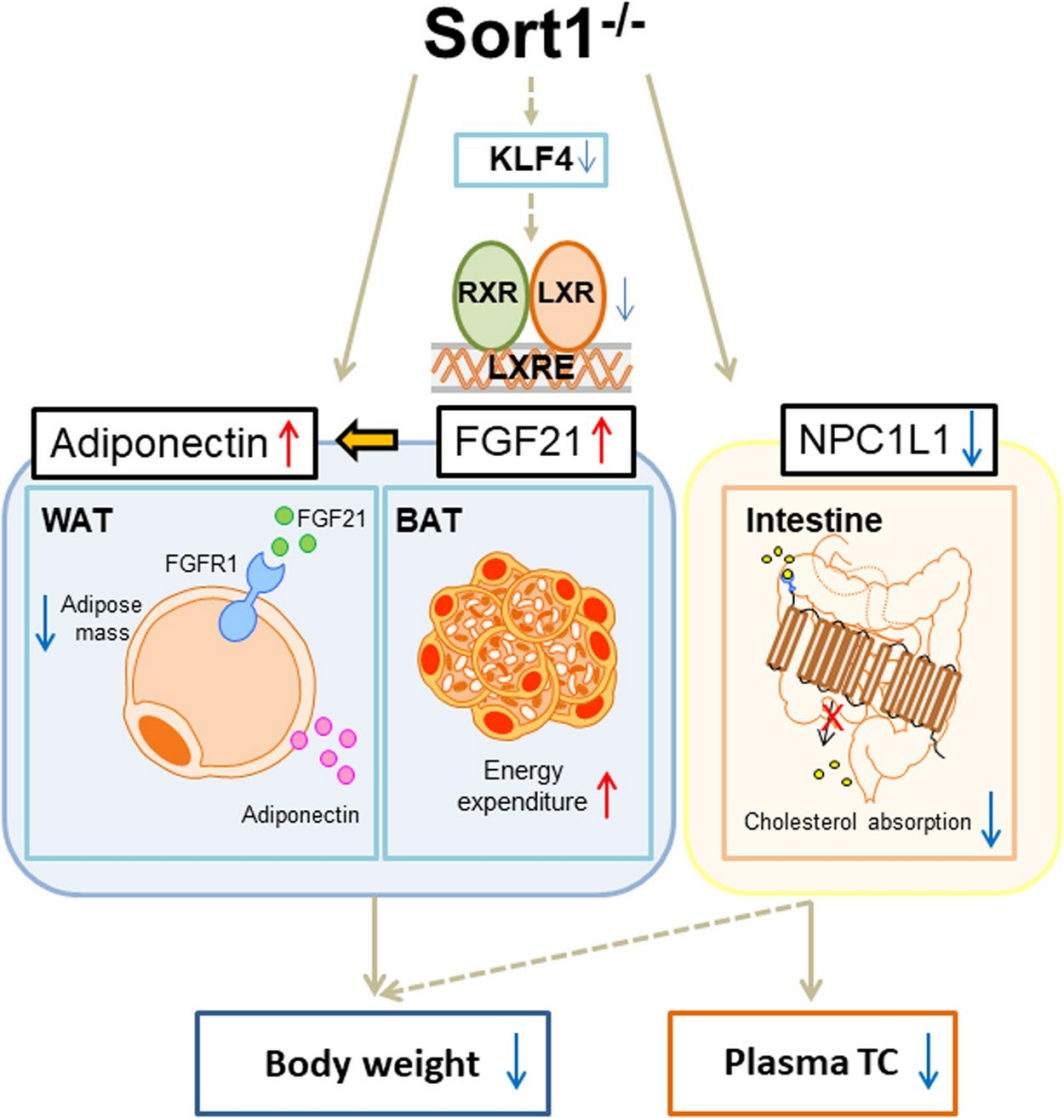
Transcriptional control of intestinal cholesterol absorption, adipose energy expenditure and lipid handling by Sortilin. Sort1 deficiency likely decreases body weight and plasma total cholesterol (TC) in female Ldlr^−/−^ mice via a KLF4-LXR signaling axis leading to decreased NPC1L1, and increased FGF21 and Adiponectin that together regulates white adipocyte formation, energy expenditure in BAT, and intestinal cholesterol absorption. Arrows indicate directionality. Dashed arrows indicate a plausible connection between KLF4 and LXR in select tissues, and that reduced NPC1L1 may partially regulate body weight via oxysterol mediated LXR-signaling.

We report the following novel findings: (1) Sort1 deficiency reduces body and WAT weight, adipocyte lipid droplet size, and increases BAT function in HF/HC-fed female Ldlr^−/−^ mice, (2) Sort1 deficiency increases FGF21 and Adiponectin in HF/HC-fed female Ldlr^−/−^ mice, (3) Sort1 deficiency decreases Npc1l1 mRNA levels and cholesterol absorption in female Ldlr^−/−^ mice and human Caco-2 cells, and 4) Sort1 deficiency decreases Klf4 mRNA and LXR-mediated transcription in female Ldlr^−/−^ mice and human cell lines. Based on our results we present the following working model (Fig. 7): In HF/HC-fed female Ldlr^−/−^ mice, Sort1 deficiency reduces LXR-mediated transcription, possibly in part via reduction of Klf4 mRNA levels in specific cell types, as exhibited by a lack of Klf4 mRNA levels change in intestinal tissue and cells. Transcriptional suppression of LXR leads to elevated FGF21 in BAT. Increased FGF21 increases BAT energy expenditure and induces Adiponectin release in WAT that lowers WAT mass and body weight gain. Suppression of LXR via Sort1 deficiency reduces Npc1l1 mRNA levels that in turn lowers cholesterol absorption. Conversely, reduced NPC1L1 may also act to lower body weight through LXR by suppressing oxysterol production. Together these mechanistic actions lower plasma TC and body weight, and associate Sortilin as a novel regulator of cardiometabolic function in female atherogenic mice.

Sortilin has been previously associated with cardiovascular disease, and metabolic and adipose tissue functions^13–16,18–23,37^; however, the connection of Sortilin between atherosclerosis and obesity, including its mechanistic role has not been established. The nuclear receptor, LXR reduces atherosclerotic lesion when activated in several models, but can also induce hepatic steatosis^38,39^. Linking cardiovascular disease with metabolic disease, LXR regulates lipid metabolism gene expression, and mice deficient in LXR have reduced HF diet-induced adipocyte size and body weight^40–42^. The mechanistic reason for Sort1 deficiency regulating female but not male LXR-related transcription in 15-week HF/HC-fed Ldlr^−/−^ mice remains to be elucidated; however, sex-specific regulation via LXR exists in mice. While LXR deficiency reduces body weight in both male and female mice, LXR deficiency exhibits sex-specific metabolic effects in female mice^43^. LXR activation reduces atherosclerosis in several mouse models, whereas LXR deficiency reduces body weight in mice; therefore, the mechanisms behind improved cardiovascular pathology and reduced body weight observed in Sort1-deficient Ldlr^−/−^ mice likely involve multiple pathways. The full mechanism of how Sort1 deficiency reduces LXR-mediated transcription in female mice and certain human cell lines requires further investigation beyond the scope of the present study; however, we identified a likely mechanistic link with Sort1 deficiency reducing the LXR-regulator, Klf4 *in vitro* and *in vivo* in examined cell types.

Connected to WAT regulation, our results support the involvement of increased energy expenditure in the suppression of female Ldlr^−/−^Sort1^−/−^ weight gain via improved BAT function. In agreement with our finding of reduced LXR-mediated transcription and increased BAT function in HF/HC-fed female Ldlr^−/−^Sort1^−/−^ mice adipose tissue, LXR induction decreases BAT function^42,44–46^. BAT regulation of energy expenditure and obesity involves several genes that had increased mRNA levels in female Ldlr^−/−^Sort1^−/−^ mice, including Ucp1, Dio2, Prdm16, and Bmp8b^45–50^. BAT can also act as a secretory tissue, including secreting FGF21. FGF21 acts on lipid metabolism and weight, is negatively regulated by LXR, and induces secretion of the obesity-associated adipokine, Adiponectin^27,31,51^.

Further tying cardiovascular pathology to energy expenditure and metabolism, we found reduced plasma TC, Npc1l1 mRNA levels, and cholesterol absorption in Sort1-deficient female Ldlr^−/−^ mice and human Caco-2 cells. In agreement with our findings, Npc1l1 deficiency or ezetimibe treatment likely protects against diet-induced obesity in mice via increased energy expenditure^52^. LXR signaling regulates the expression of lipid absorption and efflux genes (Npc1l1 and Abc-gene family members)^32^. LXR agonists increase intestinal cholesterol absorption via Npc1l1 induction^53^. On the other hand, the NPC1L1 inhibitor, ezetimibe, attenuates LXR signaling pathways via reduced cholesterol absorption and oxysterol production^54,55^. Together these studies support the notion that NPC1L1 can both regulate and be regulated by LXR. As such, it is possible that Sort1 deficiency may control body weight in female atherogenic mice, at least in part via regulation of Npc1l1 mRNA levels.

## Methods

### Mice

Low-density lipoprotein receptor-deficient mice (Ldlre/e, B6.129S7-Ldlrtm1Her/J; stock #002207) were purchased from Jackson Laboratory (Bar Harbor, ME, USA) and crossed to Sortilin-deficient mice (Sort1^−/−^) generated by targeted deletion of 191 base pairs of exon 14 (genOway, Lyon, France)^22^ to create Ldlr^−/−^Sort1^+/+^ and Ldlr^−/−^Sort1^−/−^ mice. 10-week-old male and female littermates were fed normal chow (NC) or high-fat/high cholesterol (HF/HC) (21% fat and 1.25% cholesterol, Research Diets #D12108C, New Brunswick, NJ, USA) diet for 15 weeks. Body weight and food consumption were monitored weekly, and blood was collected from the submandibular vein prior to the start of and at 5, 10, and 15 weeks on the NC and HF/HC diets. Mice were pentobarbital euthanized after 15 weeks on the study diet, and blood, peri-gonadal white adipose tissue (WAT), intrascapular brown adipose tissue (BAT), and jejunum was collected. Plasma total cholesterol (TC), triglycerides (TG), and glucose levels were assessed using Wako Pure Chemical Industries kits (Osaka, Japan; Cholesterol E-test, Triglyceride E-test, Glucose CII-test) according to the manufacturer’s protocols. Hepatic and fecal TC and TG levels were measured by chloroform:methanol (2:1) extraction in combination with the Wako TC/TG kits. Plasma Adiponectin and Fibroblast growth factor 21 levels were measured by ELISA according to the manufacture’s protocol (R&D systems, Minneapolis, MN). For histology, WAT and BAT were embedded in optimum cutting temperature compound and 7 mm serial sections were cut. Tissue samples were stained with hematoxylin and eosin (H&E). Images were captured with a digital camera (DS-Fi1c, Nikon, Melville, NY, USA) and adipocyte size was quantified using Image J software (National Institutes of Health, Bethesda, MD) as previously described^56^. All animal experiments were approved by and performed in compliance with the Institutional Animal Care and Use Committee at Beth Israel Deaconess Medical Center (protocol #010-2016).

### Cell culture

Isolated WAT and BAT was minced, and then incubated in digestion solution (Dulbecco’s Modified Eagle’s Medium (DMEM), Thermo Fisher Scientific, Waltham, MA, USA) with 5% bovine serum albumin (BSA, Sigma Aldrich, St Louis, MO, USA) and 3.3 mg/mL of Collagenase Type I (Worthington Biochemical Corp., Lakewood, NJ, USA)) for 60 minutes at 37 °C. The cell suspension was filtered through a 100 μm cell strainer (Corning, Corning, NY, USA) followed by centrifugation (1350 rpm for 10 min). The remaining pellet was suspended with ACK red blood cell lysing buffer (Thermo Fisher Scientific) and centrifuged again. After centrifugation, the cell pellet was re-suspended in DMEM containing 10% fetal bovine serum (FBS, Thermo Fisher Scientific)), 50 μg/mL Gentamicin (Corning), 50 U/mL penicillin, and 50 g/mL streptomycin (Corning), and incubated in 5% CO_2_ incubator. After reaching confluency, pre-adipocytes isolated from WAT were treated with adipogenic medium (DMEM containing 10% FBS, gentamicin, penicillin, streptomycin, 25 nM insulin, 1 nM Triiodo-L-thyronine (T3, Sigma Aldrich), 0.5 mM 3-isobutyl-1-methylxanthine (IBMX, Sigma Aldrich), 1 μM dexamethasone (Sigma Aldrich), 1 μM Rosiglitazone (Sigma Aldrich), 500 μM palmitic acid (Sigma Aldrich)). After two days, cells were cultured in insulin medium containing 25 nM insulin, 1 nM T3, 1 μM dexamethasone and 500 μM palmitic acid for 5 to 6 days. Primary pre-adipocytes isolated from BAT were treated with induction medium (DMEM with 10% FBS, gentamicin, penicillin, streptomycin, 200 μM Ascorbic acid (Sigma Aldrich), 25 nM insulin (Sigma Aldrich), 1 nM T3, 0.5 mM IBMX), 1 μM dexamethasone (Sigma Aldrich), 0.25 mM indomethacin (Sigma Aldrich)). After a day in induction media, cells were cultured in DMEM with 10% FBS, gentamicin, penicillin, streptomycin, 200 μM Ascorbic acid, 25 nM insulin, 1 nM T3, 0.25 mM indomethacin, 1 μM CL316243 (Thermo Fisher Scientific) for 5 to 6 days.

Caco-2, Hek293, and HepG2 cells were obtained from ATCC (Manassas, VA, USA). Caco-2 cells were cultured in DMEM with 10% FBS, 1% penicillin and streptomycin, and 1% Non-Essential Amino Acids Solution (NEAA, Thermo Fisher Scientific). Hek293 cells were cultured in DMEM with 10% FBS, 1% penicillin and streptomycin. HepG2 cells were cultured in Eagle’s Minimum Essential Medium (ATCC, Manassas, VA, USA) containing 10% FBS, 1% penicillin and streptomycin. RNA silencing was performed as described previously^22^. Briefly, 50 nM siRNA against Sort1 (L-010620, ONTARGETplus SMART-pool, Thermo Fisher Scientific) and non-targeting siRNA (ON-TARGET Non-Targeting Pool, Thermo Fisher Scientific) were transferred into cells using Dharmafect 4 (Thermo Fisher Scientific) or Lipofectamine RNAiMAX (Thermo Fisher Scientific) according to manufacturer’s protocols. RNA interference was performed over a 72-hour period. For LXR agonist experiments, LXR agonist treatment was performed by incubating cells in the final 24 hours of the 72-hour period with 1 μM of the LXR agonist T0901317 in DMSO (synthesized and HPLC purified by Kowa Company LTD, Tokyo, Japan).

### Intestinal cholesterol absorption

Caco-2 cells were seeded in DMEM containing 10% FBS, 1% penicillin and streptomycin, and 1% NEAA, at 4 × 10^5^ cells per well in a 12-well plate. Between 15 and 21 days after confluency, differentiated cells were washed with PBS twice and pre-incubated in serum-free DMEM for 16 hours. After pre-incubation, cells were washed with transportation buffer (140 mM NaCl, 5.4 mM KCl, 1.8 mM CaCl_2_, 0.8 mM MgSO_4_, 5 mM Glucose, 25 mM Tris (pH 7.5)) and incubated in DMEM with 5% lipoprotein-deficient serum (Sigma Aldrich) supplemented with ezetimibe (Selleck Chemicals, Houston, TX, USA) or dimethyl sulphoxide (DMSO, control) for 1 hour. After 1 hour, cells were incubated with lipid micelles (5 mM Taurocholate (Sigma Aldrich), 0.5 mM oleic acid (Sigma Aldrich), 0.04 mM phosphatidylcholine (Sigma Aldrich), 0.16 mM lysophosphatidylcholine (Sigma Aldrich), 0.3 mM mono-olein (Sigma Aldrich)), and 10 μM NBD-cholesterol (Thermo Fisher Scientific) for 4 hours. After incubation, cells were washed with PBS containing 5 mM taurocholate twice and lysed using 0.2 N NaOH with 1% SDS. Cell lysates were measured for fluorescence at excitation 465 nm and emission 535 nm, and values were normalized by protein concentration using bicinchoninic acid (BCA) protein assay kit (Thermo Fisher Scientific). For *ex vivo* cholesterol absorption assay, jejunum was harvested from Ldlr^−/−^Sort1^+/+^ or Ldlr^−/−^Sort1^−/−^ mice. Isolated tissues were cut to expose the surface using flat sections, and then cultured on 12-well plates with serum-free DMEM for 16 hours. After pre-incubation, tissues were washed with transfer buffer, and incubated with DMEM containing 5% lipoprotein-deficient serum with or without ezetimibe for 1 hour. Lipid micelles, including NBD-cholesterol were added to the culture medium and incubated for 4 hours. Tissues were homogenized using a bead-based homogenizer and NBD-cholesterol fluorescence was measured with fluorescent values normalized to total protein content.

### RNA and protein analysis

Total RNA was extracted from tissue and cells using Trizol reagent (Invitrogen, Waltham, MA, USA) according to the manufacturer’s instructions. cDNA was synthesized using a qScript cDNA Synthesis Kit (Quanta, Beverly, MA, USA). Quantitative real-time PCR was performed with commercially obtained Taqman probes (Thermo Fisher) on a 7900HT fast real-time PCR system (Applied Biosystems, Carlsbad, CA, USA). mRNA relative expression was determined by normalization to Gapdh using the delta-delta CT method. Cell protein was isolated and processed in a similar manner to which we have previously reported^57^. Briefly cells were washed with PBS and scraped from plates in RIPA buffer (Thermo Fisher Scientific) containing protease and phosphatase inhibitors. Protein content was determined by BCA assay, and 20 μg of total protein lysate was processed by SDS-PAGE and transferred onto nitrocellulose membranes using the iblot 2 apparatus (Thermo Fisher Scientific). Human cell line Sortilin was detected using an anti-Sortilin antibody (R&D Systems #AF3154) and GAPDH (Santa Cruz, #sc-47724, Dallas, TX, USA) was used as a loading control. Adipose tissue protein was isolated in RIPA buffer (Thermo Fisher Scientific) containing protease and phosphatase inhibitors, in a similar manner to which we have previously reported^37^. Briefly tissues were homogenized using a plastic pestle and run through a syringe prior to centrifuging at 5000 g for 5 minutes to pellet insoluble tissue. Supernatant protein content was quantified by BCA assay and 100 μg of total protein lysate was processed by SDS-PAGE. Mouse adipose Sortilin was detected with an anti-Sortilin antibody (Abcam #ab16640, Cambridge, UK) and GAPDH was used as a loading control.

### Graphing and statistical analysis

Data are presented as mean ± SEM; n indicates the number of samples or independent experiments performed. Statistical analyses were performed using GraphPad Prism Version 5 (Prism Software, Inc., La Jolla, CA, USA). For comparison between two groups, t-test were performed using Prism. For comparison among multiple groups, one-way ANOVA followed by post hoc tests were performed using Prism. P values less than 0.05 were considered significant. Heat maps were generated by quantitative PCR obtained mRNA levels using Qlucore software (Lund, Sweden). The working model was generated using Microsoft PowerPoint and the Motifolio illustration tool kit (Ellicott City, MD).

### Data availability

All data generated or analyzed during this study are included in this article.

## Electronic supplementary material

Supplementary Figures
