# Supplementary material for: Transcriptional control of intestinal cholesterol absorption, adipose energy expenditure and lipid handling by Sortilin
Source: Sci Rep. 2018 Jun 13;8:9006. doi: 10.1038/s41598-018-27416-y (PMC5998044; doi:10.1038/s41598-018-27416-y)
Supplement: Supplementary file 1 — Supplementary Figures [file 41598_2018_27416_MOESM1_ESM.pdf]

Transcriptional control of intestinal cholesterol absorption, adipose energy expenditure and lipid handling by Sortilin

Sumihiko Hagita, Maximillian A. Rogers, Tan Pham, Jennifer R. Wen, Andrew K. Mlynarchik, Masanori Aikawa, and Elena Aikawa

Data Supplement

	Male NC $Ldlr^{-/-}Sort1^{+/+}$ (n=6)	Male NC $Ldlr^{-/-}Sort1^{-/-}$ (n=8)
Food consumption (g/day)	6.02 ± 1.52	6.04 ± 1.22
Liver/BW ratio (mg/g BW)	47.27 ± 1.99	42.41 ± 0.54
Plasma glucose (mg/dL)	228.01 ± 18.93	229.25 ± 15.64
Hepatic triglyceride (mg/g liver)	37.04 ± 9.31	25.77 ± 3.22
Hepatic total cholesterol (mg/g liver)	2.70 ± 0.20	2.52 ± 0.17
	Male HF/HC $Ldlr^{-/-}Sort1^{+/+}$ (n=6-8)	Male HF/HC $Ldlr^{-/-}Sort1^{-/-}$ (n=9-11)
Food consumption (g/day)	2.59 ± 0.09	2.57 ± 0.09
Liver/BW ratio (mg/g BW)	58.97 ± 2.37	57.60 ± 2.03
Plasma glucose (mg/dL)	222.43 ± 12.10	224.31 ± 13.40
Hepatic triglyceride (mg/g liver)	127.00 ± 22.13	83.08 ± 13.51
Hepatic total cholesterol (mg/g liver)	11.84 ± 1.07	8.39 ± 1.33 *
	Female NC $Ldlr^{-/-}Sort1^{+/+}$ (n=9)	Female NC $Ldlr^{-/-}Sort1^{-/-}$ (n=8)
Food consumption (g/day)	5.87 ± 0.79	5.98 ± 0.91
Liver/BW ratio (mg/g BW)	41.17 ± 0.86	43.69 ± 0.62 *
Plasma glucose (mg/dL)	183.96 ± 30.66	199.10 ± 29.21
Hepatic triglyceride (mg/g liver)	42.23 ± 5.09	35.55 ± 9.10
Hepatic total cholesterol (mg/g liver)	3.32 ± 0.21	4.15 ± 0.60
	Female HF/HC $Ldlr^{-/-}Sort1^{+/+}$ (n=8-10)	Female HF/HC $Ldlr^{-/-}Sort1^{-/-}$ (n=7-10)
Food consumption (g/day)	2.36 ± 0.14	2.33 ± 0.18
Liver/BW ratio (mg/g BW)	65.55 ± 2.57	59.89 ± 2.65
Plasma glucose (mg/dL)	211.94 ± 10.14	205.10 ± 14.16
Hepatic triglyceride (mg/g liver)	109.62 ± 24.42	81.98 ± 8.29
Hepatic total cholesterol (mg/g liver)	18.62 ± 2.51	16.58 ± 1.56

Supplemental Table S1. 15-week fed NC and HF/HC-fed $Ldlr^{-/-}Sort1^{+/+}$ and $Ldlr^{-/-}Sort1^{-/-}$ mice characteristics. *P < 0.05 versus sex and diet matched $Ldlr^{-/-}Sort1^{+/+}$ mice, analyzed by t-test; values are presented as mean ± SEM.

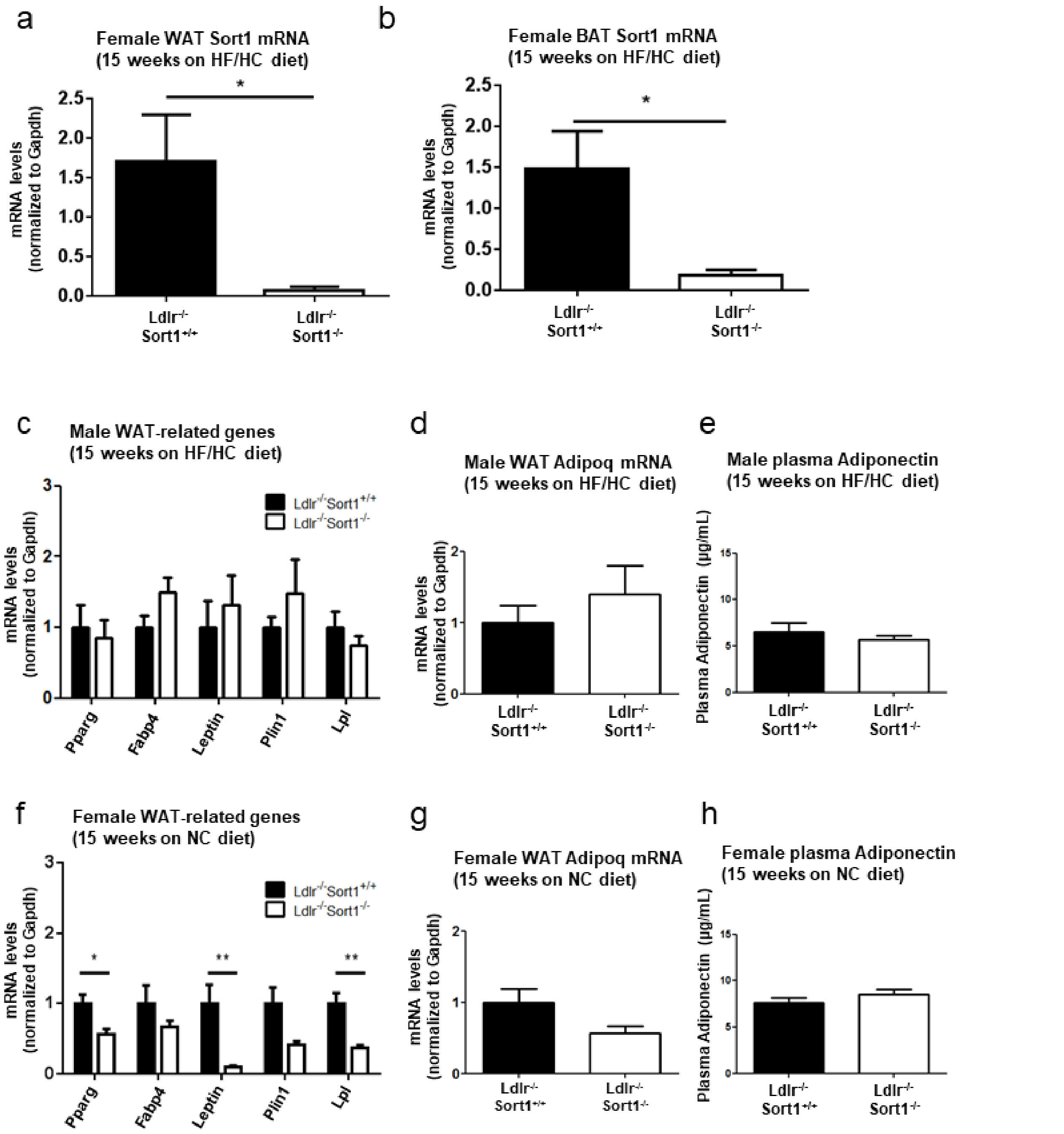

Supplemental Figure S1. Sortilin deficiency did not alter WAT in HF/HC-fed male Ldlr$^{-/-}$ mice. (**a**) Sort1 deficiency mRNA levels confirmation in WAT and (**b**) BAT of female Ldlr$^{-/-}$ mice fed a HF/HC diet for 15 weeks (n=5 mice/group). (**c**) WAT white adipose-related gene mRNA levels (n=4-7 mice/group), (**d**) WAT Adipoq mRNA levels (n=4-7 mice/group), and (**e**) plasma Adiponectin protein abundance in 15-week HF/HC-fed male Ldlr$^{-/-}$ mice (n=5-6 mice/group). (**f**) WAT white adipose-related gene mRNA levels (n=4-6 mice/group), (**g**) WAT Adipoq mRNA levels (n=5 mice/group), and (**h**) plasma Adiponectin protein abundance in 15-week NC-fed female mice (n=5 mice/group). *P < 0.05, **P < 0.01 versus Ldlr$^{-/-}$Sort1$^{+/+}$, analyzed by t-test; values are presented as mean ± SEM.

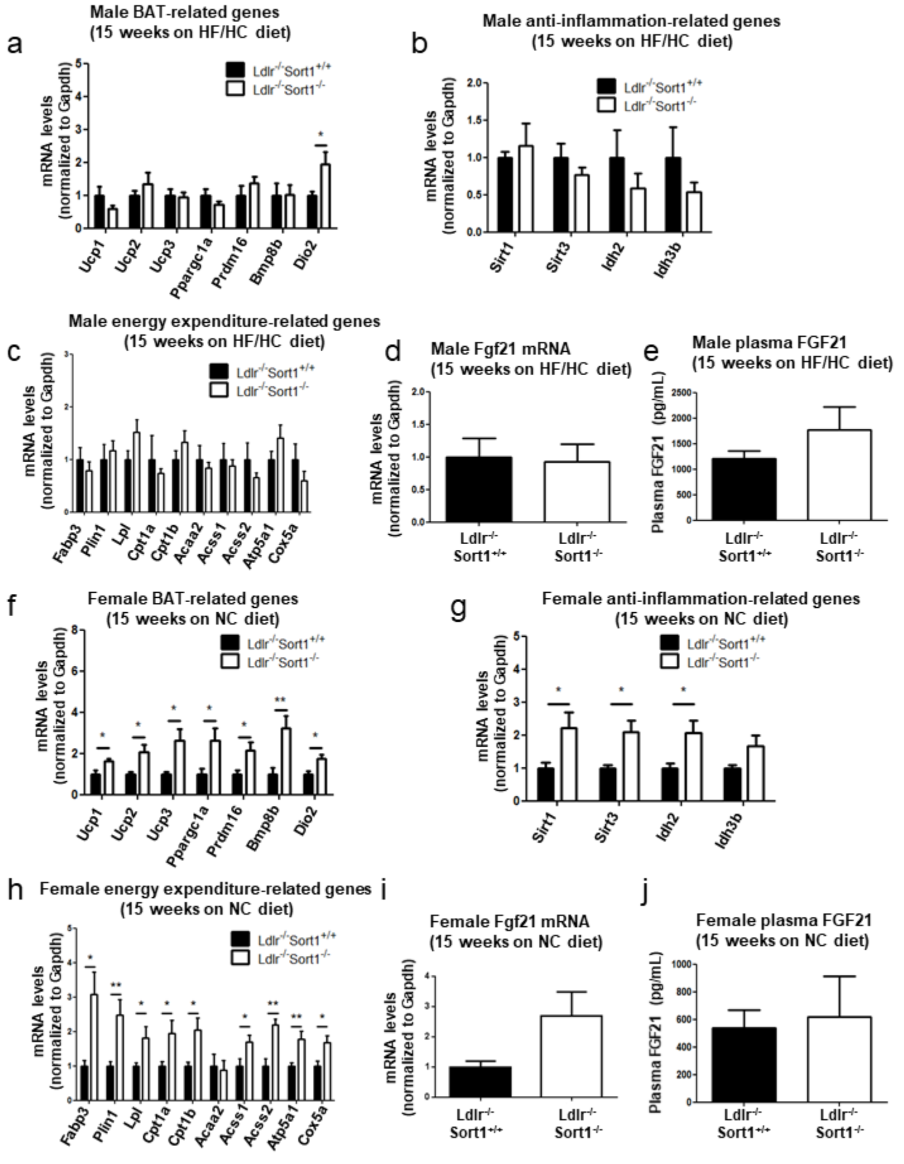

Supplemental Figure S2. Sortilin deficiency did not alter plasma FGF21 in HF/HC-fed male Ldlr$^{-/-}$ mice. (**a**) BAT brown adipocyte-related, (**b**) anti-inflammation-related, and (**c**) energy expenditure-related (including fatty acid utilization, β-oxidation, TCA cycle and electron transport chain) mRNA levels in 15-week HF/HC-fed male mice (n=4-6 mice/group). (**d**) BAT Fgf21 mRNA levels (n=3-4 mice/group), and (**e**) plasma FGF21 protein abundance (n=7-8 mice/group) in male Ldlr$^{-/-}$Sort1$^{+/+}$ and Ldlr$^{-/-}$Sort1$^{-/-}$ mice fed a HF/HC diet for 15 weeks. (**f**) BAT brown adipocyte-related (n=4-6 mice/group), (**g**) anti-inflammation-related (n=5-6 mice/group), and (**h**) energy expenditure-related expression (n=5-6 mice/group) in 15-week NC-fed female mice. (**i**) BAT Fgf21 expression (n=4-6 mice/group), and (**j**) plasma FGF21 protein abundance (n=7-9 mice/group) in female Ldlr$^{-/-}$Sort1$^{+/+}$ and Ldlr$^{-/-}$Sort1$^{-/-}$ mice fed a NC diet for 15 weeks. *P < 0.05, **P <0.001 versus Ldlr$^{-/-}$Sort1$^{+/+}$ mice, analyzed by t-test; values are presented as mean ± SEM.

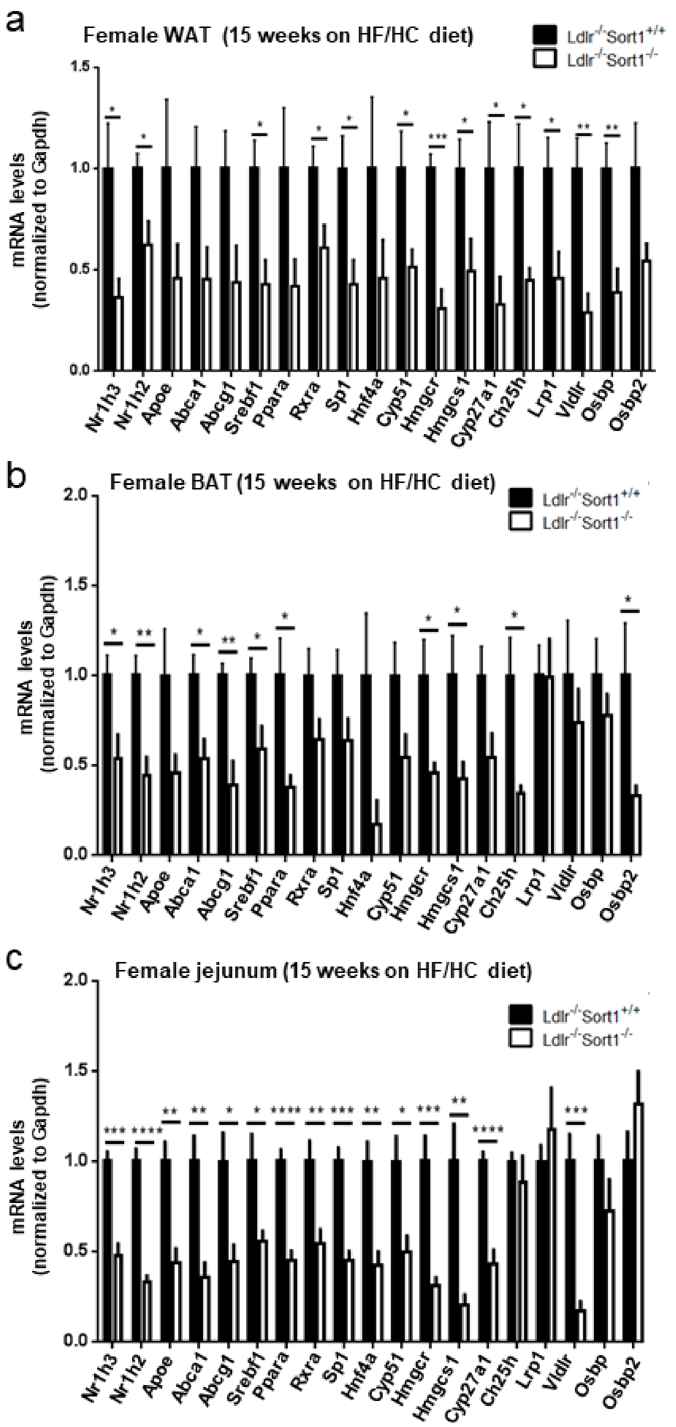

Supplemental Figure S3. Sortilin deficiency suppressed LXR-related transcription in female Ldlr$^{-/-}$ mouse WAT, BAT, and jejunum. LXR and sterol-associated gene mRNA levels in (**a**) WAT, (**b**) BAT, and (**c**) jejunum of female Ldlr$^{-/-}$Sort1$^{+/+}$ and Ldlr$^{-/-}$Sort1$^{-/-}$ mice fed a HF/HC diet for 15 weeks; (n=5-6 mice/group). *P < 0.05, **P < 0.01, ***P < 0.001, ****P < 0.0001 versus Ldlr$^{-/-}$Sort1$^{+/+}$, analyzed by t-test; values are presented as mean ± SEM. mRNA levels data is also presented as a heat map in Figure 5.

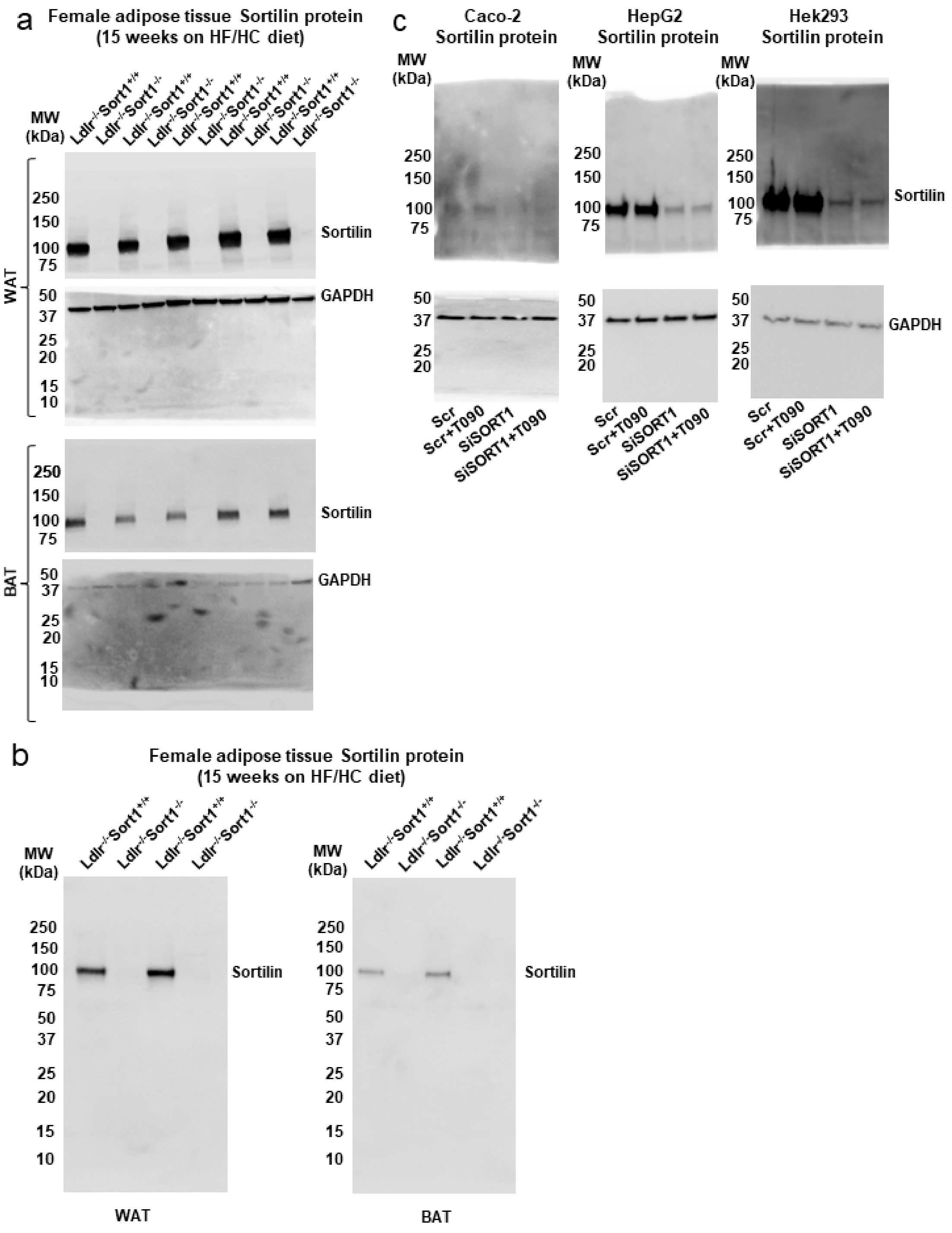

Supplemental Figure S4. Full-length blots. (**a**) Full-length blots for Fig. 1a; GAPDH loading controls were run on the same gel, and the membrane was cut prior to incubating with primary antibody. (**b**) 15 weeks HF/HC diet-fed female mouse WAT and BAT tissue uncut full-length Sortilin western blots. (**c**) Full-length blots for Fig. 6a; GAPDH loading controls were run on the same gel, and the membrane was cut prior to incubating with primary antibody. Molecular weight (MW) kilodalton (kDA) ladder indicated on the side of blots.
